# Thermo-Optical Switching Effect Based on a Tapered Optical Fiber and Higher Alkanes Doped with ZnS:Mn

**DOI:** 10.3390/ma13215044

**Published:** 2020-11-09

**Authors:** Joanna E. Moś, Karol A. Stasiewicz, Katarzyna Matras-Postołek, Leszek R. Jaroszewicz

**Affiliations:** 1 Faculty of New Technology and Chemistry, Military University of Technology, 2 Kaliskiego St., 00-908 Warsaw, Poland; karol.stasiewicz@wat.edu.pl (K.A.S.); leszek.jaroszewicz@wat.edu.pl (L.R.J.); 2 Faculty Chemical Engineering and Technology, Cracow University of Technology, 24 Warszawska St., 31-155 Cracow, Poland; katarzyna.matras-postolek@pk.edu.pl

**Keywords:** tapered optical fiber, thermo-optical switcher, alkanes, optical fiber sensor, nanomaterials

## Abstract

The paper investigates the effect of thermo-optic switching resulting from the hybrid combination of a tapered optical fiber (TOF) with alkanes doped with nanoparticles of zinc sulfide doped with manganese (ZnS:Mn NP). Presented measurements focused on controlling losses in an optical fiber by modification of a TOF cladding by the alkanes used, characterized by phase change. Temperature changes cause power transmission changes creating a switcher or a sensor working in an ON-OFF mode. Phase change temperatures and changes in the refractive index of the alkane used directly affected power switching. Alkanes were doped with ZnS:Mn NPs to change the hysteresis observed between ON-OFF modes in pure alkanes. The addition of nanoparticles (NPs) reduces the difference between phase changes due to improved thermal conductivity and introduces extra nucleating agents. Results are presented in the wide optical range of 550–1200 nm. In this investigation, hexadecane and heptadecane were a new cladding for TOF. The higher alkanes were doped with ZnS: Mn NPs in an alkane volume of 1 wt.% and 5 wt.%. The thermo-optic effect can be applied to manufacture a thermo-optic switcher or a temperature threshold sensor.

## 1. Introduction

**Figure 1:**
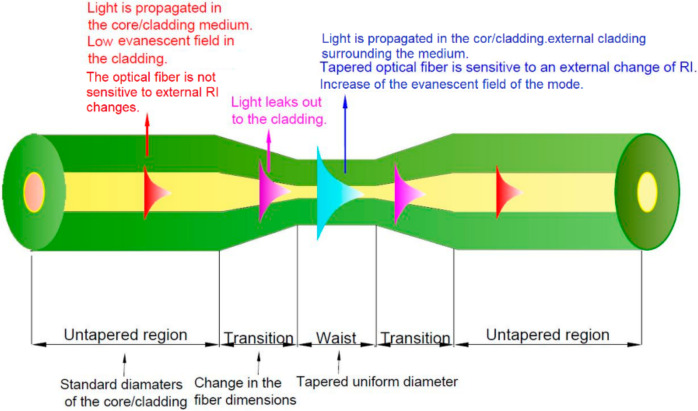
Graphic presentation of boundary conditions changes and modification of light propagation along the regions of a tapered optical fiber.

Refractive index (RI) is one of the materials basic optical parameters. This parameter value is determined by physical, chemical, and optical factors like temperature, humidity, pressure, wavelength, concentration of chemical compounds, etc. [1,2,3]. RI measurement, with one of these parameters changings, allows for the creation of optical device solutions [4,5]. In this paper, the thermo-optic effect is based on the phase change of alkanes (solid–liquid state), which is related to the material reflective index (RI) variation due to temperature changes. The basic optical and sensitive to RI parameters changes element is a tapered optical fiber (TOF) which provides interaction of a electro-magnetic light wave with an external surrounding medium [6,7,8]. Penetration depth of the evanescent field (EF) in a TOF is the most important phenomenon due to its possible application in a tapered fiber in optical devices such as switchers, sensors, filters, etc. Fiber tapering increases the EF light in a TOF cladding and enables its interaction with external surroundings in the taper waist region [9]. In this case, the boundary conditions for the propagated light are changed. Figure 1 shows the graphic presentation of this phenomenon. Modes propagated in the optical fiber are modified along the TOF length (characteristic regions—transition and taper waist). In point A, the optical fiber dimension does not change and light is propagated in the core/cladding medium based on a total internal reflection. The light on the boundary between two media shows a continuous field distribution. Hence, it is necessary to consider the existence of EF penetrating the cladding and showing an exponential decay with an increasing distance from the core/cladding interface. In point B, the fiber diameters decrease (transition regions of TOF). The result of the tapering of the fiber structure is an increase in the power propagated outside the core as EF. The light beam is reflected more frequently. In point C, the light is propagated in a core/cladding external surrounding medium. TOF is sensitive to RI external changes, due to the large EF [10,11,12].

EF depends on a few factors: RI of the core (n_core_), RI of the surrounding medium—cladding (n_cladding_), TOF radius (a decreased taper radius provides the EF increase, light propagated as EF determines sensitivity of a sensor based on TOF), and operational wavelength (λ). The penetration depth (d_p_) of EF is given by [9,13]:

(1) $$d_{p}=\frac{\lambda }{2\pi \sqrt{n_{\mathrm{core}}{}^{2}{sin}^{2}\theta_{i}-n_{\mathrm{cladding}}{}^{2}}},$$

where θ_i_ is the angle of incidence of a plane wave on the core/cladding interface.

This EF effect observed in TOF allows using an extra material in the taper surroundings forming a double clad structure [14,15,16]. Changes in the RI of the external material change the optical properties of the light propagated in an optical fiber structure. Combination of a functional material and TOF has been used to build, more sensitive and specialized in measuring different parameters, optical devices like sensor, filter, attenuator, etc. Selection of external materials plays an important part in creating sensitive sensors. The material should be chosen in terms of optical parameters (RI, low optical losses) and considering a possible use of its special properties (phase changes, anisotropies, absorption, etc.), allowing to change the material optical properties affecting the propagated light and interacting with the measured factor. For example, a liquid crystal (LC) and its anisotropic properties (ordinary and extraordinary RI) could be controlled by electric field and magnetic field, and changes in these coefficients could be detected at the same time. Additionally, the LC structure depends on temperature. If we change the temperature, the LC refractive indices are lower, which influences the light propagation [17]. Different optical configurations with LC are used as thermo-switch, electro-switch, or optical sensor [18]. In general, a different optical fiber configuration is applied as a thermo-optical switcher including couplers, Mach-Zehnder interferometers, filled photonic crystal fibers, polyimide waveguides, etc. [19,20,21,22,23,24,25,26]. An optical switching effect may be obtained using an optical fiber element (as TOF) and a phase change material (PCM) as alkane [26,27]. 

In this paper, a TOF-based thermo-optical switching with a modified cladding through the use of alkanes doped with nanoparticles of zinc sulfide with manganese is presented. The alkanes used are characterized by a phase change that determines this effect, which can be applied as a threshold sensor of temperature or a thermo-optic switcher for different optical wave ranges depending on the used material: pure alkanes or a mixture of alkanes and ZnS: Mn NPs.

## 2. Materials and Methods 

### 2.1. Alkanes as Phase Change Materials (PCM)

Alkanes are organic chemical compounds consisting of carbon and hydrogen. The number of carbon atoms (m) determines number of hydrogens according to the formula C_m_H_2m+2_ [28]. Generally, as carbon atoms increase (and their molecular weight), the melting point and heat of alkanes increase. This is due to higher intermolecular interactions. Alkanes are stable during thermal cycles (solid–liquid and liquid–solid state)—lack of phase segregation [29,30]. The phase segregation is an undesirable effect for practical applications. In the case of an inorganic material, this effect is counteracted by adding a liquid viscosity-increasing substance to this material. In organic materials like alkanes, phase transmissions take place over a wide temperature range (phase changes hysteresis). The latent heat of fusion influences the thermal hysteresis effect. The temperature range is also affected by the rate of temperature changes (which is connected with material thermal conductivity) [31]. To understand the physical nature of the phase change hysteresis, one needs to analyze changes in the internal energy of material during the heating and cooling process. During the heating process, the material heats up and its temperature rises. At some point (upon obtaining the melting point), despite further heating, the material’s temperature remains constant and the crystal phase changes by changing the material structure. Energy that must be delivered to the material is defined as a latent heat. Only after this energy is delivered to the material, the alkane temperature increases again. Materials with a high latent heat value are used in heat storage applications like a phase change material (PCM). Only after the phase transformation process is completed, this material temperature increases further in the entire volume [32]. In the reverse cooling process, the heat is transferred outside (temperature limit in this case is a solidification temperature, which is equal to melting temperature). During the cooling process, the number of crystallization centers in the liquid is also an important parameter determining the process speed. In the paper, we described the situation where adding NPs to alkanes allows for a heterogeneous crystallization process which, in turn, is to accelerate the cooling process of the material around TOF. 

As mentioned above, alkanes belong to the group of PCMs [29,30,31,32,33,34]. Their most important parameters are thermal conductivity and heat capacity (depending on specific heat and latent heat). Thermal conductivity describes an effective heat exchange between the system and the environment. Alkanes thermal conductivity is in the range of 0.15–0.30 W/mK [29]. It is a low value of this parameter in relation to an inorganic material. To improve thermal conductivity of alkanes, these materials are doped with materials of good conductivity. Graphite, metals (gold NPs), and oxides (aluminum oxide) are examples of doping materials [22,35,36]. In this article, the NPs ZnS: Mn was used to improve thermal conductivity and influence thermal hysteresis. Heat capacity provides ability to material to accumulate heat. Alkanes heat capacity is in the range of 152–269 kJ/kg [29]. For these materials, the latent heat of fusion is a very important parameter during the phase change process. Alkanes melting heat can be modified by mixing different alkanes [29].

In this paper, transmission changes through TOF are investigated. Transmission changes depend on changes of alkanes RI, which create an additional cladding material surrounding TOF and change the boundary condition of the propagated light beam. Alkanes are characterized by step discontinue changes of RI during the phase change, which is widely described in literature [28,37]. Alkanes melting point determines changes in the alkane RI value. In general, alkanes phase-transitions determine their optical transparencies for light propagated in TOF. The light in TOF can be transmitted only when alkane is in a liquid state and its refractive index is lower than the optical fiber RI. If temperature is lower than the melting point, alkanes are in the solid state and are not transparent. If we analyze a complex refractive index of alkanes, in solid state, the extinction ratio is high [26], which causes light scattering and, eventually, light full attenuation. 

RI, melting point, and thermal parameters of other alkanes described above determine the threshold operation of the thermo-optic effect with thermal hysteresis for cooling and heating process in the proposed transducers.

### 2.2. Properties of Used Alkanes and TOF Technology

**Figure 2:**
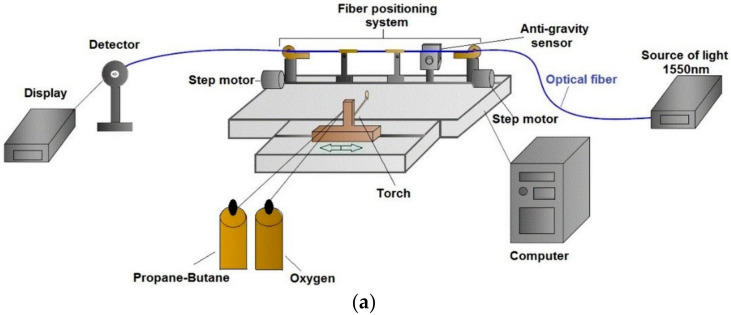

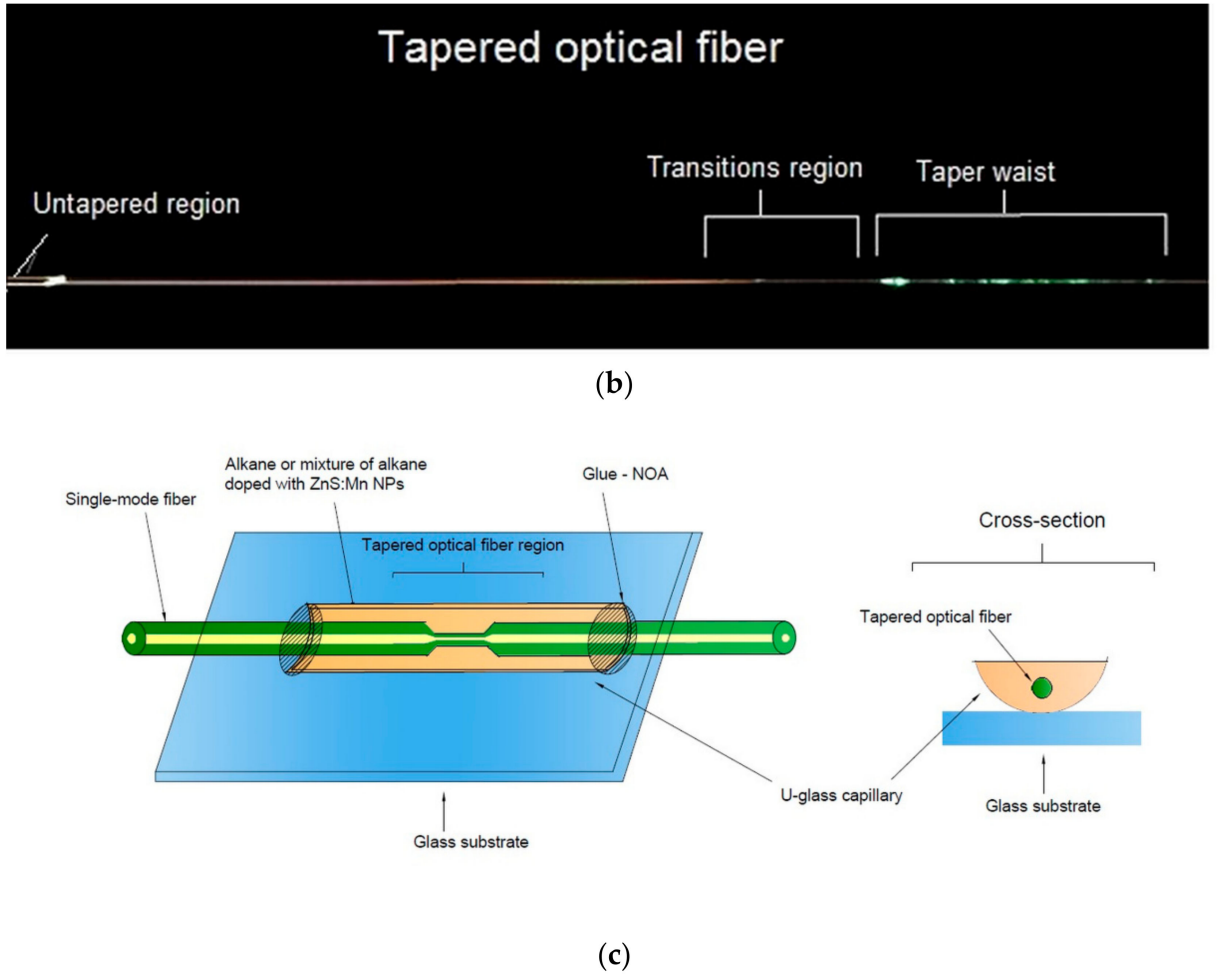
(**a**) Scheme of a set-up for preparing a tapered optical fiber, (**b**) tapered optical fiber manufactured on Fiber Optic Taper Element Technology (FOTET), and (**c**) scheme of the prepared sample for a transmission measurement.

This part of the paper presents the TOF technology, the sample preparation with additional materials, and the main properties of the materials used. TOF was fabricated on a FOTET station (Fiber Optic Taper Element Technology)-(Figure 2a) [17,27,38]. FOTET has used a movable torch with a mixture of gases, propane–butane and oxygen, to heat the optical fiber optic section to a suitable temperature. The mixing ratio of gases is set using gas regulators. This part of technology is important because it determines the flame temperature, which directly influences the elongation process. If temperature is too low, the tapering process stops. Otherwise, if it is too high, the optical fiber melts. Optical fibers were uniformly elongated by step motors, after obtaining an appropriate softening temperature. Elongation velocity was controlled by the software connected with an anti-gravity sensor. Fiber height was regulated by this sensor. The sensor prevented “falling” on the burner and breaking the optical fiber. Manufactured TOFs were characterized by losses below 0.5 dB@1550 nm, elongation l = 28.71 ± 0.16 mm, taper waist diameter d = 6.0 ± 0.5 µm, and torch movement of 5 mm (Figure 2b). Tapers were made with a single-mode fiber (SMF). This optical fiber is characterized by a core diameter of 8.2 µm, a cladding diameter of 125 ± 0.7 µm, and a cut-off wavelength < 1260 nm made by Corning [39]. This paper presents changes in light propagation. Tests were carried out in a wide range of wavelengths of 550–1200 nm. For this range, the SMF fiber used works as a multimode fiber. The selected range was dictated by a device operation verification for a wavelength range other than the second and third telecommunication window. Preliminary studies have been carried out for the range of 1200–1800 nm for TOF and pure alkanes. As a result, the optical device working at wavelengths of 1200–1800 nm with low loss was presented [27]. Research showed that it is possible to build the optical switch based on TOF and alkanes in a different optical range [27]. Additionally, the research in this paper was extended to describe influences of NPs on the phase change in alkanes and a direct impact of such materials on the light propagation in TOF. The manufactured TOF was secured by the U-glass capillary (Figure 2c). The capillary at the same time protected the taper against mechanical damage and also enabled the permanent connection of TOF with fixed volume alkanes (Figure 2c). Prepared samples were tested in several thermal cycles (heating-cooling process). The manufactured TOF was used only once for a given material (pure alkane or alkane +1 wt.% NPs or alkane +5 wt.% NPs).

In this paper, two higher alkanes: hexadecane (C_16_) and heptadecane (C_17_), pure and doped with 1 wt.% and 5 wt.% of ZnS:Mn nanoparticles were investigated as a TOF external medium. Table 1 presents the selected thermal, physical, and optical properties of pure alkanes described in this article. C_16_ is characterized by a lower melting temperature compared to C_17_. As presented in Table 1, both alkanes have similar refractive indices in a liquid state. The reflective indices in a liquid state are lower than the RI of a single-mode optical fiber (≈1.46 [39]). In general, changes of a new TOF cladding RI depend on temperature determining alkane phase transitions. In a liquid state, the materials were characterized by a lower RI than in the optical fiber taper enabling the light propagation with low losses (ON mode). The light leaked out of the taper structure when alkanes were in a solid state (OFF mode). TOF losses increased in alkane solid state because the increased extinction ratio caused light leaking into the new cladding. Therefore, the important parameter determining changes in ON-OFF mode is the melting temperature of the selected alkanes. Alkanes latent heat is an effective energy storage during the phase transition process. This also has a direct impact on magnitude of the phase change hysteresis. C_16_ is characterized by a higher value of this parameter. Kinematic viscosity and specific heat of the selected materials are also shown in Table 1. 

C_16_ and C_17_ are characterized by a low thermal conductivity. In this paper, tests of mixtures of alkanes and nanoparticles (NP) ZnS: were provided to enhance their thermal conductivity, as well as to introduce heterogeneous nucleation centers created by NPs. The intended effect of this combination was the acceleration of phase changes (melting and solidification heat transfer rates) and, as a result, the reduction in the phase change hysteresis occurring for pure alkanes (ON-OFF mode in thermo-optical switching). ZnS:Mn NPs were prepared at the Cracow University of Technology, Cracow, Poland. ZnS:Mn NPs had diameters of about 10 nm [44]. Compounds of ZnS:Mn are characterized by RI in the range of 2.29–2.53 [45]. The thermal conductivity of zinc sulfide is of 16.7 W/m·K [46]. In order for nanoparticles to be added to other materials, it is needed to functionalize nanocrystals. System of capping agents like cysteaminium chloride combined with a 4-dodecylbenzenesulfonic acid was applied [47]. To prevent an agglomeration of nanoparticles, a special material Brij78p-polyethylene glycol monooctadecyl ether was used. This material did not influence optical properties of the mixture of alkanes and NPs. Thanks to the tests carried out on pure alkanes and mixture alkanes with a different ratio of ZnS:Mn NPs, changes of light transmission in TOF depending on a temperature variation could be observed.

**Figure 3:**
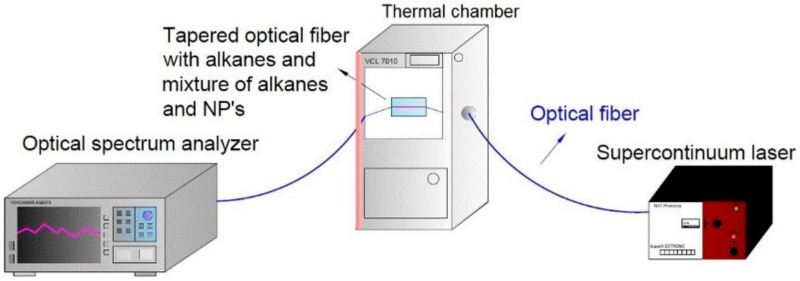
Scheme of a set-up for measuring the transmission of a tapered optical fiber depending on changes of temperature.

In Figure 3, the optical measurement system is presented. Main devices used during the measurement were as follows: supercontinuum white light laser model SuperK Extreme (NKT Photonics, Southampton, UK), optical spectral analyzer model AQ6373 (Yokogawa, Tokyo, Japan), and thermal chamber model VCL 7010 (Votsch Industrietechnik, Balingen, Germany). The thermal chamber allows controlling the speed of changes in temperature. The uniform distribution of ZnS:Mn NPs in the mixture was obtained by the shaker IKA (Vortex 1, Staufen, Germany).

**Figure 4:**
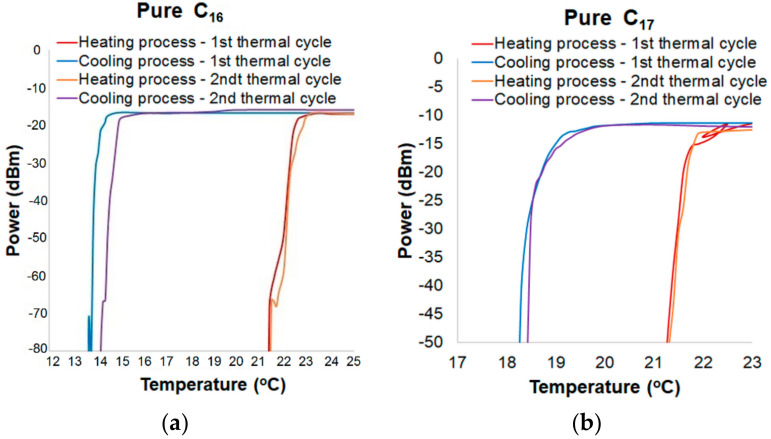
Changes of power transmission in tapered optical fiber (TOF) with an extra material for two thermal cycles for the measured samples: (**a**) pure C_16_ and (**b**) pure C_17_.

Temperature changes were at a rate of 0.5 °C/3 min. This function of the thermal chamber ensures results repeatability. Alkanes are a stable material in thermal cycles for the same rate of temperature changing (Figure 4a,b).

## 3. Results

**Figure 5:**
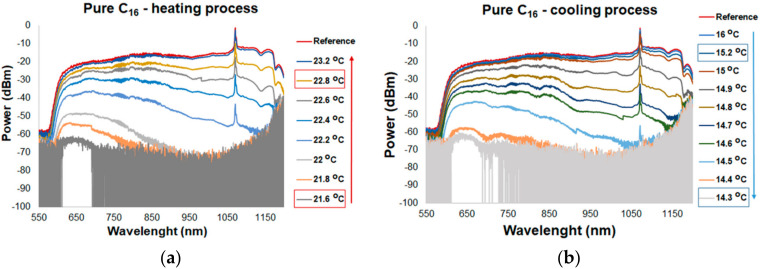

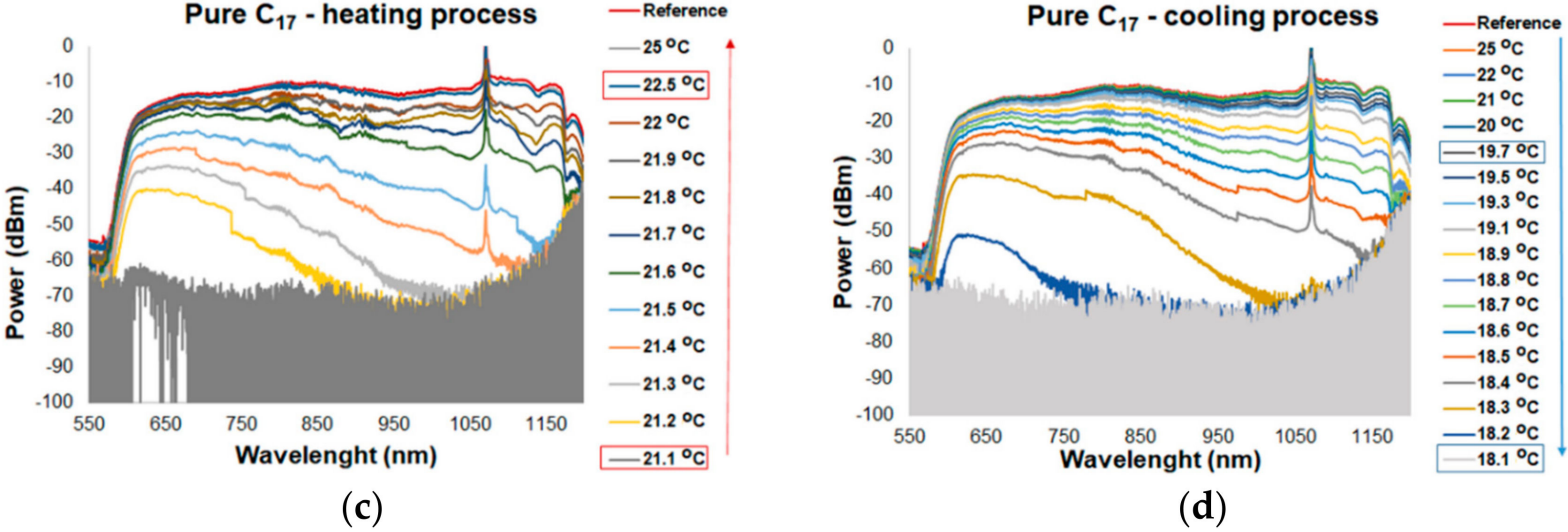
Transmission of a tapered optical fiber in the range of 550–1200 nm for different temperatures for: (**a**) heating process for pure C_16_, (**b**) cooling process for pure C_16_, (**c**) heating process for pure C_17_, and (**d**) cooling process for pure C_17_.

Figure 5 shows the spectral characteristics of TOF with pure C_16_ and C_17_ for the heating and cooling process. Measurements for a hybrid combination of TOF with pure alkanes were a reference to the transmission changes for TOF with alkanes doped with ZnS:Mn NPs for different concentrations and temperatures. The changes in the spectral characteristics were directly influenced by the change in the RI of the alkane, as well as the melting and freezing temperature. Following the data from Table 1, the melting temperature of C_16_ is of 18 °C. ON mode (transmission of light) was of around 21.6 ± 0.3 °C while it was heated (Figure 5a). At this temperature, alkane RI decreased to the level at which light could be propagated by the TOF with no loss. In general, C_16_ in a liquid state is transparent to light and the reference level (TOF without alkane) is the same as for a transducer with alkane. RI of C_16_ is lower than RI of TOF. This provides excellent conditions for light propagation on a total internal reflection. For each cycle of heating and cooling, the loss was the same. For the heating process after exceeding the melting point, the range of a wavelength transmission started from a shorter wavelength and was expanded with increasing temperature. The penetration depth had a direct impact on the transmission changes. As the wavelength increases, the depth of penetration increases, which, in turn, leads to an increase in the interaction of light with the environment. Additionally, for the chosen wave range and fiber properties, an internal mode interference observed as an oscillation of the light propagation was obtained. The effect was observed for each of the samples with pure alkanes and mixtures of alkanes with ZnS:Mn NPs. Temperatures difference when changing transmission from a narrow wavelength range to transmission over the entire wavelength range is of about ΔT_expand_ ≈ 1.1–1.5 °C (Figure 5a), and it changes during the research. When TOF with C_16_ is cooled, OFF mode is within temperatures of 14.3 ± 0.3 °C (Figure 5b). At this temperature, alkanes are in a solid state, i.e., the extinction ratio is increased, and the material is not transparent to light. Propagated light leaks out to the cladding (absorption/scattering). Changing transmission effect depending on a wavelength is similar to the heating process. Decrease in the transmission is observed from longer wavelengths to shorter ones. Temperature difference with variable transmission over the entire measuring wavelength range until no transmission is of about ΔT_narrow_ ≈1–1.5 °C. Transducer based on TOF and the pure C_17_ temperature response is shown in Figure 5c,d. C_17_ melts at a temperature of 22 °C. Temperature at ON mode is of around 21.1 ± 1 °C. For this transducer, ΔT_expand_ ≈1.5 °C. The slower power changes shown in the spectral characteristics for C_17_ are the result of the fact that C_17_ has a lower thermal conductivity than C_16_. Temperature at OFF mode is of 18.1 ± 0.1 °C. For this transducer, ΔT_narrow_ ≈1.5 °C.

**Figure 6:**
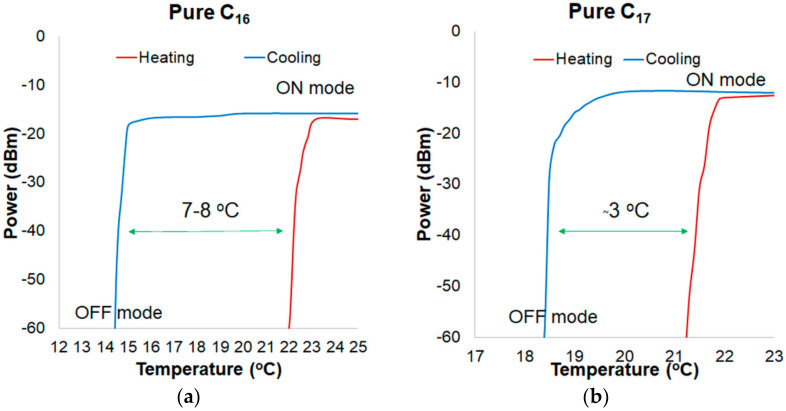
Hysteresis of power changing for a wavelength of 810 nm for (**a**) pure C_16_ and (**b**) pure C_17_.

In a graph showing the hysteresis for alkanes, the heating process was plotted in red and the cooling process was in blue. Figure 6a presents the hysteresis of power changing for a transducer based on C_16_ and TOF for a wavelength of 810 nm. Temperature difference between the ON-OFF mode is of around 7–8 °C. Hysteresis occurrence results from the thermal properties of alkanes, such as thermal conductivity and latent heat. In Figure 6b, the hysteresis of power change for a wavelength of 810 nm for pure C_17_ is presented. Temperature difference between heating and cooling processes is of about 3 °C. Compared to the transducer with pure C_16_, the hysteresis for pure C_17_ is lower. It is a result of lower latent heat of C_17_ (data from Table 1, as well as the melting point). In addition, it should be noticed that for C_17_ alkane in a cooling mode, the power of light decreases in a much smoother and slower way than in C_16_. It is a result of a higher molecular weight of C_17_, which is strictly connected with numbers of C groups and intermolecular interactions.

**Figure 7:**
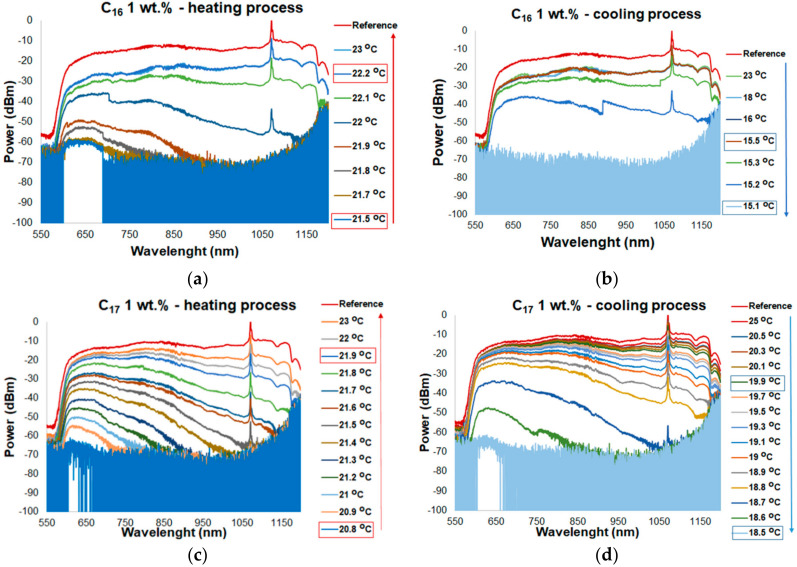
Transmission of a tapered optical fiber in the range of wavelengths of 550–1200 nm for different temperatures for: (**a**) heating process for C_16_ + 1 wt.% ZnS:Mn nanoparticles (NPs), (**b**) cooling process for pure C_16_ + 1 wt.% ZnS:Mn NPs, (**c**) heating process for pure C_17_ + 1 wt.% ZnS:Mn NPs, and (**d**) cooling process for pure C_17_ + 1 wt.% ZnS:Mn NPs.

Figure 7 shows the spectral characteristics of a transducer based on TOF with a mixture of alkanes C_16_ and C_17_ doped with 1 wt.% ZnS:Mn NPs for heating and cooling process. For C_16_, the ON mode was around a temperature of 21.5 ± 0.2 °C when it was heated see on Figure 7a. The ON state temperature was similar to a transducer with a pure C_16_, but the increase in a transmission for the entire wavelength measurement range accelerated by 0.5. That result confirms the assumption: NPs were heterogeneous nucleation centers and increased thermal conductivity of alkanes. In Figure 7b, the spectral characteristics for the cooling process are showed. The OFF mode was of around 15.1 ± 0.2 °C. Changing the transmission for this sample was very fast—parameter ΔT_narrow_ is of about 0.3 °C. Taking into account Figure 7, the reference level is higher than power when the mixture of C_16_ and 1 wt.% is in a liquid state. The increased transmission loss can be the result of a deposition of NPs on TOF. ON mode for a transducer with C_17_ doped with 1 wt.% ZnS:Mn NPs is of around 20.8 ± 0.2, OFF mode is of around 18.5 + 0.1 °C (see on Figure 7c). ΔT_expand_ is equal to ≈1.1 °C, which is 0.4 °C less than for the pure alkane, and ΔT_narrow_ is of about 1.3 °C, which is 0.2 °C less than for the pure material (Figure 7d).

**Figure 8:**
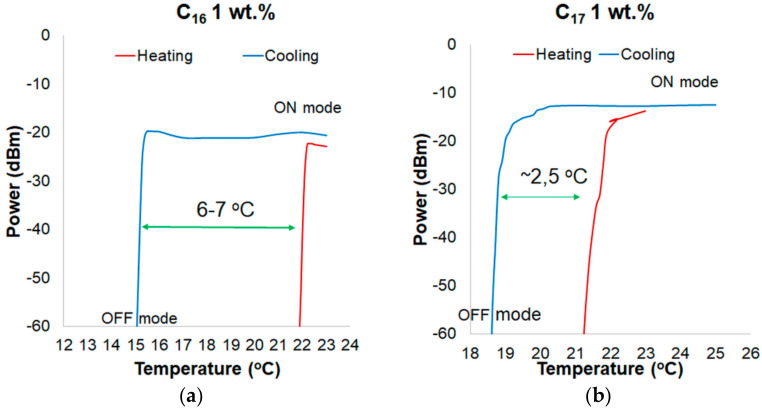
Hysteresis of power changing for a wavelength of 810 nm for: (**a**) pure C_16_ and (**b**) pure C_17_.

In Figure 8a, a hysteresis of power change for a wavelength of 810 nm for C_16_ with 1 wt.% ZnS:Mn NPs is presented. Compared to the transducer with pure C_16_, the hysteresis was reduced to 6–7 °C. It is less of about 1 °C compared to the pure material. In Figure 8b, a hysteresis for a wavelength of 810 nm for C_17_ with 1 wt.% ZnS:Mn NPs is shown, which was reduced to 2.5 °C. It is about 0.5 °C lower than in pure alkane. 

**Figure 9:**
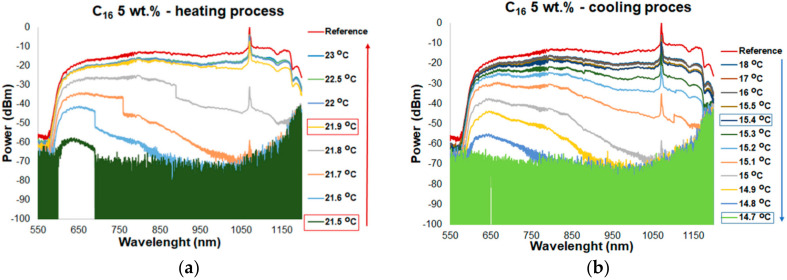

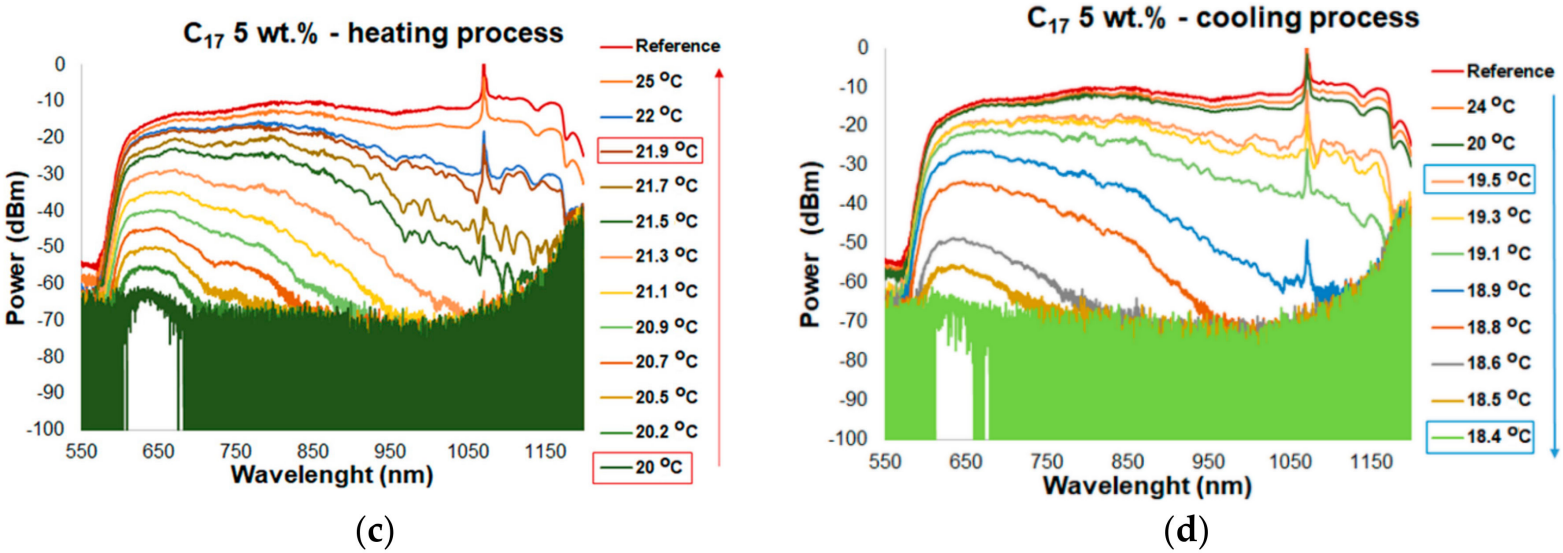
Transmission of a tapered optical fiber in the range of 550–1200 nm for different temperatures for: (**a**) heating process for C_16_ + 5 wt.% ZnS:Mn NPs, (**b**) cooling process for pure C_16_ + 5 wt.% ZnS:Mn NPs, (**c**) heating process for pure C_17_ + 5 wt.% ZnS:Mn NPs, and (**d**) cooling process for pure C_17_ + 5 wt.% ZnS:Mn NPs.

Figure 9a,b for C_16_ with 5 wt.% of NPs presents changes of power for the heating–cooling processes, respectively. The heating process was accelerated compared to the transducer with pure alkanes (ΔT_expand_) of about 0.5 °C. On the spectral characteristics for the heating process (see on Figure 9a), the acceleration of phase changes is visible as a step change of the transmission line for a given temperature in the temperature range of the phase change of this material. Disturbances in the spectral characteristics are the result of collecting data over a wide range of wavelengths when the material surrounding TOF alters the physical properties. The ON mode was of around 21.5 ± 0.2 °C. The OFF mode for the cooling process was of around 14.8 ± 0.2 °C (show in the Figure 9b). The transmission was changing in the same way as in a transducer with pure alkane but with an increased rate of solidification from about ΔT_narrow_ ≈1 °C (C_16_) to ΔT_narrow_≈ 0.5 °C (C_16_ + 5 wt.%). The ON mode for C_17_ with 5 wt.% of NPs was of around 20 + 0.2 °C and OFF mode was of 18.4 + 0.1 °C (Figure 9c,d). In this case, also the phase-change acceleration process was observed. ΔT_expand_ was reduced to 1 °C and ΔT_narrow_ to 1 °C. This was about 0.5 °C less than for a pure material for the heating and cooling process.

**Figure 10:**
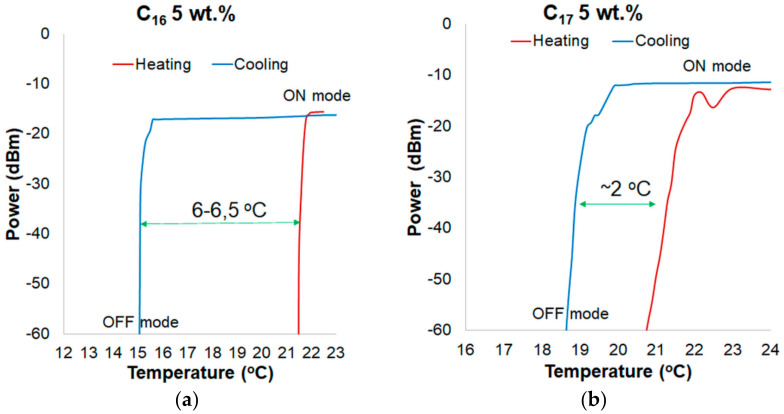
Hysteresis of power changing for a wavelength of 810 nm for: (**a**) pure C_16_ and (**b**) pure C_17_.

In Figure 10a,b, a hysteresis of power change for a wavelength of 810 nm for C_16_ and C_17_ with 5 wt.% ZnS:Mn NPs is presented. Compared to the transducer with pure C_16_, and C_16_ + 1 wt.% ZnS:Mn NPs, the hysteresis was reduced to 6–6.5 °C. For C_17_, the hysteresis was reduced to 2 °C. 

**Figure 11:**
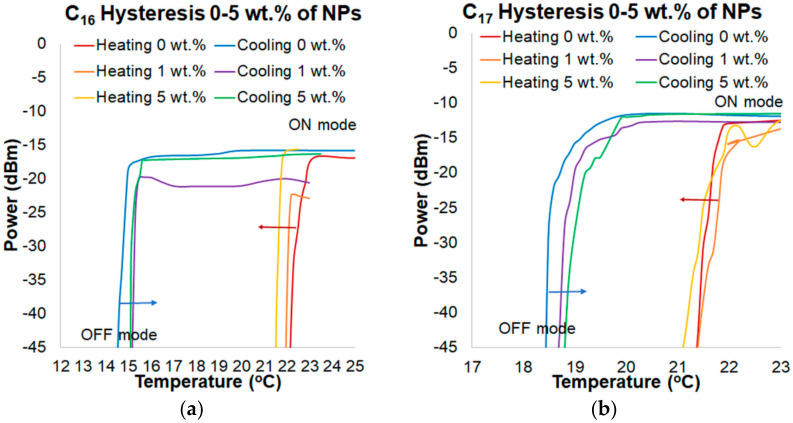
Hysteresis of power changing depending on temperature for a wavelength of 810 nm for: (**a**) C_16_ with pure alkane and alkane with doping ratio of 1 wt.% and 5 wt.% of ZnS:Mn NPs and (**b**) C_17_ with pure alkane and alkane with doping ratio of 1 wt.% and 5 wt.% of ZnS:Mn NPs.

In general, for a transducer with C_16_, the hysteresis was reduced to around 1.5 °C (for 5 wt.% compared to pure C_16_) (Figure 11a). Temperature of the ON mode for the heating process was reduced with an increasing doping of NPs. Temperature of the OFF mode for the cooling process was also changed. Curves of the cooling process were shifted to a higher temperature with an increasing doping of NPs. Process of heating and cooling was accelerated by doping ZnS:Mn NPs. The addition of NPs improves thermal properties of alkane. Hysteresis of transducers with C_17_ was reduced to around 1 °C for 5 wt.% compared to C_17_ (Figure 11b). Curves of the heating process were shifted to a lower temperature with an increasing doping. The OFF mode temperature for the cooling process was also changed. In this case, in the same way as in transducers with C_16_, the process of heating and cooling was accelerated by doping ZnS:Mn NPs.

In Table 2, the collective measurement results for TOF-based transducers with pure C_16_ and C_17_ and, also, alkanes doped with different ratio of ZnS: Mn NPs are presented.

In general, pure alkanes were characterized by a lower RI in the liquid state than RI of the TOF material. The power was comparable to the reference (TOF without additional material). Low power losses for transducers of TOF with mixtures of alkanes and NPs are the result of NPs deposition on TOF. Due to the alkanes doping with ZnS:Mn NPs, a reduction in the phase change hysteresis was achieved. The addition of NPs accelerated the phase transformation processes. Transmission in the whole measuring range of a wavelength was achieved faster for transducers doped with ZnS:Mn NPs. The best expected effect was obtained for 5 wt.% of NPs. Adding ZnS:Mn NPs reduces alkane hysteresis due to the improved thermal conductivity and introduces extra nucleating agents. If we analyzed the spectral characteristics for all measurements, the switching ON-OFF mode for different wavelengths is different. The switching temperatures increase with increasing wavelengths. It is a result of a different penetration depth of wavelengths. During the process of phase change, TOF detected an effective reflective index, which can be the mean value of RI for liquid and solid state. When the material is melted in the whole volume, the light propagation with low loss is possible for a whole range of wavelengths. 

The next research step is to check the hysteresis change for mixtures of alkanes characterized by a lower heat of melting than pure alkanes. The second task is to obtain a precise control over the ON-OFF switching mode for given temperatures. Presented solutions of a hybrid combination of TOF and alkanes or alkanes with ZnS:Mn NPs provide us with a good opportunity to create a low cost, simple thermo-optical switcher or a temperature threshold sensor.

## 4. Conclusions

The research presented in the article focuses on changes in the boundary conditions for TOF with the use of additional higher alkanes materials and mixtures of alkanes and ZnS:Mn NPs. This operation changes the properties of the light (loss) propagated in the optical fiber as a result of a change in the temperature used for thermo-optic switching. The article presents the possibility of controlling the light transmission in a wide range of wavelengths. Depending on the alkanes used, as well as the degree of doping with nanoparticles, we can obtain fast thermo-optical switching devices operating in the ON-OFF mode dedicated to selected applications. The use of ZnS:Mn NPs changes the properties of materials, such as heat capacity and thermal conductivity, and introduces new crystallization centers that affect temperature changes in the ON-OFF mode. As noticed, the increase in ZnS:Mn NPs concentration caused decreased differences between ON-OFF modes. In all cases, the antiagglomeration material Brji78 was used to obtain an even distribution of NPs in a material volume. The research shows the possibility of using a combination of TOF and higher alkanes or mixtures of alkanes with NPs for threshold sensors or thermo-optic switches. Advantages of the created transducer are: economy, small size, simplicity, and stability. The device operates in the wide range of wavelengths (550–1200 nm) for each of the materials used in the study. Depending on the selected higher alkanes, the device can work for different temperature ranges. Additionally, applying different kinds of NPs, it is possible to shift hysteresis in a controlled way.

## Figures and Tables

**Table 1:** Properties of the higher alkanes used [33,40,41,42,43].

Symbol	Alkane	Melting Point (°C)	n (20 °C, 589.3 nm)	Thermal Conductivity (W/mK)	Latent Heat (kJ/kg)	Specific Heat (kJ/kg)	Kinematic Viscosity (mm^2^/s)
C_16_	n-hexadecane (C_16_H_34_)	18	1.4345	0.2	237	2.078	(20 °C) 4.51
C_17_	n-heptadecane (C_17_H_36_)	22	1.4369	0.145	213	2.078	(40 °C) 3.47

**Table 2:** Results for tested transducers based on tapered optical fiber (TOF) and higher alkanes with different doped ratio of ZnS Mn nanoparticles (NPs).

	C_16_	C_16_ + 1 wt.% ZnS:MnNPs	C_16_ + 5 wt.% ZnS:MnNPs	C_17_	C_17_ + 1 wt.% ZnS:MnNPs	C_17_ + 5 wt.% ZnS:MnNPs
Hysteresis (°C, 810 nm)	7–8	6–7	6	3	2.5	2
ΔT_expand_ (°C) *	1.1–1.5	0.8–1	0.5	1.5	1.1.	1
ΔT_narrow_ (°C) **	1–1.5	0.3–0.4	0.5	1.5	1.3	1

* Temperature difference at which obtained transmission is in a whole range of measurement wavelengths, point of starting process is T_ON_. ** Temperature difference at which there is no transmission in a whole range of measurement wavelengths, point of finishing process is TOFF.
